# Editorial: Physical Education among gifted students

**DOI:** 10.3389/fspor.2025.1565121

**Published:** 2025-02-10

**Authors:** Alejandro Prieto-Ayuso, Sebastiaan Willem-Jan Platvoet, Mark de Niet, Álvaro Infantes-Paniagua

**Affiliations:** ^1^ Faculty of Education of Cuenca, Department Physical Education, Arts and Music, University of Castilla-La Mancha, Cuenca, Spain; ^2^ HAN University of Applied Sciences, Nijmegen, Netherlands; ^3^ Faculty of Education of Albacete, Department Physical Education, Arts and Music, University of Castilla-La Mancha, Albacete, Spain

**Keywords:** talent identification, sports performance, talent transfer, physical characteristics, stereotypes, high ability students

**Editorial on the Research Topic**
Physical Education among gifted students

Giftedness remains a critical educational issue, as gifted and talented students are often not adequately supported in the classroom. When discussing these students, we refer to those who “perform—or have the capability to perform—at higher levels compared to others of the same age, experience, and environment in one or more domains” [(1), p.1]. Traditionally, the focus has been on their academic and intellectual development, while their physical dimension has been less addressed or even neglected in the literature (2). At this juncture, it is important to distinguish between two themes. Firstly, we can consider the physical dimensions of any gifted student, including their physical traits, fitness, and engagement in physical activity and sport. This theme encompasses their inclusion in Physical Education (PE) lessons and the potential educational adaptations required. Secondly, we can focus on students who are physically gifted or talented, with topics centered on identifying their gifts and talents and ensuring their inclusion and appropriate attention in PE lessons.

PE is particularly relevant for intellectually gifted and talented students, as they tend to exhibit lower levels of physical self-concept than their non-gifted peers (3). However, scientific literature on this topic is scarce. PE can also be highly beneficial for physically gifted students, offering them additional opportunities to support their development into future elite athletes. Whereas in many countries additional classes for those who stay behind in motor development are common, as far as we know, additional classes for gifted students are still scarce. This can also be seen in scientific literature, where there have been an absence of research on athletic identification in journals that aim to publish about high ability and education (4) that continues until our days.

Recent evidence highlights how physical activity can positively influence cognitive, intellectual, academic, emotional, and social dimensions in children and adolescents [e.g. (5)]. This makes the research topic even more pertinent within the gifted and talented domain. Therefore, we proposed the current research topic with the aim of exploring and synthesizing the latest findings from various research perspectives on this subject. In doing so we raised some questions to the research community: How can physically or intellectually gifted and talented students be identified? How do they feel about being in PE and gifted? Which instruments and programs are more adequate to be used in PE to help them develop? How can PE and sport collaborate more to support physically talented/gifted students? We finally included four high quality works within this research area that show evidence on some of these issues.

First, Jung undertook a systematic review to outline the key details of current research on the identification and development of physical giftedness/talent. The five broad emerging themes related to conceptions of physical giftedness/talent, identification characteristics/criteria, factors associated with identification, identification methods, and talent development interventions.

Second, Silva et al. focused on the effects of a multivariate training program on physical fitness and tactical performance. The results showed that in a school environment, a well-structured multivariate training program can effectively improve students' tactical skills, increasing their physical conditioning levels.

Third, Green et al. provided us with a discussion on talent transfer through a systematic review of the literature with a three-fold aim: to explore how talent transfer has been defined in order to develop a synthesized definition; to identify the factors that influence talent transfer; and to investigate how theory underpins and enhances understanding of talent transfer. The review exposes a scarcity of theoretical foundation in current research, suggesting ecological dynamics as a promising approach to advance research and practice.

Finally, Ferrándiz et al. focused on pre-service teachers, recognizing their crucial role in the future of education for gifted and talented students. The implications of this study highlight the need to further improve the content related to giftedness in teacher training programs, despite the apparently better-informed pre-service teachers in the sample. It also suggests the consideration of conducting meta-analyses to clarify the existing evidence.

Together, the four studies highlight the importance of a broad and multidimensional understanding of talent and its development in different contexts, including educational. Each research paper addresses a key aspect that contributes to the overall picture: from conceptualizing and identifying talent to preparing educators who play a critical role in supporting gifted and talented students.

In short, PE plays a fundamental role in the overall well-being of gifted and talented students, through both physical and socio-emotional development. Through structured activities and tailored programs, these students can channel their energy, improve motor coordination, sport related skills, and achieve optimal physical health. Furthermore, PE fosters skills such as teamwork, resilience, and stress management, which are crucial for their personal and social development. Therefore, it is essential to design programs that integrate challenging and enriching activities, promoting and supporting not only their gifts and talent but also a holistic well-being that enables them to thrive in all aspects of their lives.

The development of this research topic has also highlighted several limitations. The primary limitation is the scarcity of research. Despite efforts to engage researchers and authors in the fields of talent in PE and giftedness, the low number of received and ultimately included studies indicates that this topic still lacks attention. This is concerning given the significant and beneficial impact that focused research could have on physically or intellectually talented or gifted students. Furthermore, educational researchers, particularly in PE, often overlook gifted individuals. Similarly, sports researchers frequently forget the opportunities that PE and PE teachers present for identifying and developing gifted students or athletes. This paucity of research is a setback for advancing the state of the art in this area. However, we hope that by drawing attention to this issue, we can encourage the scientific and educational communities to place greater emphasis on physical activity and the role of PE in supporting gifted and talented students. Only through increased attention and inter-disciplinary research we can design, develop, validate, and ultimately implement evidence-based educational practices for all students.

Future lines of research could focus on expanding theoretical and methodological frameworks to identify and develop talent across various domains, including physical, academic, and social aspects. It would be valuable to explore interdisciplinary approaches that integrate neuroscience, psychology, and technology, such as artificial intelligence and big data, to enhance the precision in identifying and understanding talent over time. In the area of talent transfer, it would be important to investigate the factors that facilitate this transition between disciplines and contexts, incorporating multicultural perspectives and considering variables such as gender and access to resources. Teacher training should be strengthened through evidence-based interventions that include practical methodologies and differentiated approaches to address gifted students, also evaluating how teachers' beliefs and attitudes influence the development of these students. Finally, a cross-cutting area of interest would be to explore how both new technologies and digital environments can enhance talent identification and development, fostering holistic and integrated approaches that cover all stages of personal and professional growth. These are only some of the possibilities that could be developed from this area of research. However, we should not forget that, as the research on talent development points out [e.g. (6, 7)], the environmental context where students grow and develop (e.g., teachers, trainers, family, etc.) is key in the development of their talents. PE can play therefore an important role in gifted and talented students' development. More attention in PE to gifted students is not only beneficial for them but will also strengthen the importance of PE for the success of a society.
